# Advanced Pelvic Girdle Reconstruction with three dimensional-printed Custom Hemipelvic Endoprostheses following Pelvic Tumour Resection

**DOI:** 10.1007/s00264-024-06207-3

**Published:** 2024-05-22

**Authors:** Xin Hu, Minxun Lu, Yitian Wang, Yi Luo, Yong Zhou, Xiao Yang, Chongqi Tu, Li Min

**Affiliations:** 1 Department of Orthopedic Surgery and Orthopedic Research Institute, West China Hospital, Sichuan University, No. 37 Guoxue Road, Chengdu, 610041 Sichuan China; 2 Model Worker and Craftsman Talent Innovation Workshop of Sichuan Province, No. 37 Guoxue Road, Chengdu, 610041 Sichuan China; 3 National Engineering Research Center for Biomaterials, Sichuan University, Chengdu, 610064 China; 4 Provincial Engineering Research Center for Biomaterials Genome of Sichuan, Sichuan University, Chengdu, 610064 China

**Keywords:** 3D-printed, Prostheses, Hemipelvectomy, Pelvic tumour, Pelvic girdle

## Abstract

**Purpose:**

Resection of pelvic bone tumours and subsequent pelvic girdle reconstruction pose formidable challenges due to the intricate anatomy, weight-bearing demands, and significant defects. 3D-printed implants have improved pelvic girdle reconstruction by enabling precise resections with customized guides, offering tailored solutions for diverse bone defect morphology, and integrating porous surface structures to promote osseointegration. Our study aims to evaluate the long-term efficacy and feasibility of 3D-printed hemipelvic reconstruction following resection of malignant pelvic tumours.

**Methods:**

A retrospective review was conducted on 96 patients with primary pelvic malignancies who underwent pelvic girdle reconstruction using 3D-printed custom hemipelvic endoprostheses between January 2017 and May 2022. Follow-up duration was median 48.1 ± 17.9 months (range, 6 to 76 months). Demographic data, imaging examinations, surgical outcomes, and oncological evaluations were extracted and analyzed. The primary endpoints included oncological outcomes and functional status assessed by the Musculoskeletal Tumor Society (MSTS-93) score. Secondary endpoints comprised surgical duration, intraoperative bleeding, pain control and complications.

**Results:**

In 96 patients, 70 patients (72.9%) remained disease-free, 15 (15.6%) had local recurrence, and 11 (11.4%) succumbed to metastatic disease. Postoperatively, function improved with MSTS-93 score increasing from 12.2 ± 2.0 to 23.8 ± 3.8. The mean operating time was 275.1 ± 94.0 min, and the mean intraoperative blood loss was 1896.9 ± 801.1 ml. Pain was well-managed, resulting in substantial improvements in VAS score (5.3 ± 1.8 to 1.4 ± 1.1). Complications occurred in 13 patients (13.5%), including poor wound healing (6.3%), deep prosthesis infection (4.2%), hip dislocation (2.1%), screw fracture (1.0%), and interface loosening (1.0%). Additionally, all patients achieved precise implantation of customized prosthetics according to preoperative plans. T-SMART revealed excellent integration at the prosthesis-bone interface for all patients.

**Conclusion:**

The use of a 3D-printed custom hemipelvic endoprosthesis, characterized by anatomically designed contours and a porous biomimetic surface structure, offers a potential option for pelvic girdle reconstruction following internal hemipelvectomy in primary pelvic tumor treatment. Initial results demonstrate stable fixation and satisfactory mid-term functional and radiographic outcomes.

**Supplementary Information:**

The online version contains supplementary material available at 10.1007/s00264-024-06207-3.

## Introduction

The pelvic girdle is a common site for primary malignant bone tumours, representing around 15% of all primary sarcomas [1]. Advancements in imaging technologies, surgical methodologies, and chemotherapy regimens have significantly curtailed the imperative for limb amputation [2, 3]. As a result, limb salvage surgery has progressively become the standard treatment for pelvic malignancies [4, 5]. However, the intricate pelvic anatomy, often accompanied by extensive tumour invasion, presents considerable challenges in both resecting pelvic bone tumours and subsequent pelvic girdle reconstruction [6]. Moreover, various reconstruction methods, including both biological options (such as arthrodesis, hip transposition, and allograft/autograft reconstruction) [7–10] and non-biological approaches (like endoprosthetic reconstruction) [11–14], each offer distinct advantages and drawbacks, leading to variability in clinical outcomes. This diversity further complicates decision-making, given the absence of consensus on the optimal solution. Among these reconstruction options, endoprosthetic reconstruction has gained acclaim for its initial stability, satisfactory cosmetic results, and shorter hospital stays, as well as its relatively swift restoration of function [11, 12, 15, 16]. However, the use of endoprostheses is linked to a certain degree of incidence of implant loosening and infection [2, 17–19]. These factors pose limitations on its long-term functional outcomes and clinical applicability.

The emergence of three-dimensional (3D) printing has greatly improved the production of orthopaedic implants, theoretically enabling the construction of implants in any shape for customized anatomical adaptation in bone defect repairs [17, 20–27]. Furthermore, 3D-printed titanium alloy implants can incorporate surface porous structures with precise pore size parameters, facilitating bone formation and osseointegration [27]. With the remarkable benefits of 3D-printed implants, musculoskeletal oncology centers worldwide have developed customized hemipelvic endoprostheses for pelvic girdle reconstruction [28]. Numerous early clinical follow-up studies have reported superior clinical outcomes compared to traditional reconstruction methods with higher Musculoskeletal Tumor Society Score (MSTS) ratings and decreased rates of postoperative implant loosening (Table 1) [17, 18, 22, 24, 29–37].

**Table 1:** Summary of Major Studies on Pelvic Reconstruction Using 3D-Printed Custom Endoprostheses Following Tumor Resection

Study	No. of patients	Reconstruction Type	Fixation	Follow-up period (mean, months)	Functional score (mean)	Cost and time (mean, USD and days)	Major Complications
Guo et al. [18]	3	Type I/I + IV	Cancellous screws + Polyaxial Pedicle Screw	20.5	MSTS-93, 22.7	NA	None
Yu et al. [38]	10	Type I/I + IV	Cancellous screws + lumbar pedicle screws	47.0	MSTS-93, 23.9	NA/12	DWH
Hao et al. [29]	6	Type I/I + IV	Flanges and screws + cancellous screws	32.2	NA	NA/8	None
Guo et al. [18]	12	Type II/I + II/II + III/I + II + III	Cancellous screws + bone cement	20.5	MSTS-93, 19.8	NA	None
Guo et al. [18]	20	Type II/I + II/II + III/I + II + III	Cancellous screws + lumbar pedicle screws	20.5	MSTS-93, 17.7	NA	None
Tu et al. [36]	13	Type II/I + II/II + III/I + II + III	Cancellous screws + Endoprosthestic stem	27.0	MSTS-93, 23	7000/10	None
Shao et al. [17]	11	Type II/I + II/II + III/I + II + III	Cancellous screws + pubic plate and screws	15.5	MSTS-93, 19.2	NA/14	HD, DWH
Ji et al. [1]	80	Type II/I + II/II + III/I + II + III	Cancellous screws + cementation	32.5	MSTS, 25.2	NA	LR, DI, HD
Hu et al. [39]	13	Type II/I + II/II + III/I + II + III	Cancellous screws + extracortical plate and screws	22.4	MSTS, 22.0	NA/14	LM, LR, HD
Zoccali et al. [34]	14	Type II/I + II/II + III/I + II + III	Cancellous screws + extracortical plate and screws	42.0	MSTS, 13.9	12,800/NA	LR, DWH
Zhu et al. [40]	15	Type II/I + II/II + III/I + II + III	Cancellous screws	28.0	MSTS-93, 24.5	NA/7	LR, DWH,
Huang et al. [41]	16	Type II/I + II/II + III/I + II + III	Cancellous screws + extracortical plate and screws	17.8	MSTS-93, 25.7	NA	DVT, DWH, Pneumonia, HD
Broekhuis et al. [42]	15	Type II/I + II/II + III/I + II + III	Locking screws + flanges and screws	33.8	MSTS, 19.0	NA/28	HD, DI, LR
Wang et al. [32]	11	Type II/I + II/II + III/I + II + III	Cancellous screws + flanges and screws	30.0	MSTS-93, 21.4	15,000/9	HD, DI, LR
Lu et al. [43]	19	Type II/I + II/II + III/I + II + III	Cancellous screws + cementation	30.0	MSTS-93, 22.7	NA	LR, HD, AL
Hao et al. [29]	24	Type II/I + II/II + III/I + II + III	Flanges and screws + cancellous screws	32.2	MSTS-93, 23.2	NA/8	Superficial infection, HD
Donati et al. [37]	8	Type II/I + II/II + III/I + II + III	Cancellous screws + flanges and screws + pubic screw	43.4	MSTS, 27.0	NA	LR, LM
Park et al. [30]	2	Type III	Pubic plate and screws + a socket inserting the contralateral pubic ramus	13.9	MSTS-93, 30	NA/15	None
Tu et al. [31]	5	Type III	Pubic stem + cancellous screws	24.0	MSTS-93, 29.8	7000/10	ED

*DWH *delayed wound healing, *LR *local recurrence*, DI *deep infection*, ED *erectile dysfunction*, DVT *deep venous thrombosis, *LM *lung metastasis*, HD *hip dislocation*, AL *aseptic loosening

However, as a relatively young technology, the clinical application of 3D-printed hemipelvic endoprostheses has been limited by their short period of use [18, 30–36]. Additionally, pelvic malignant tumours, being relatively rare, result in a restricted number of cases available to individual medical institutions. Therefore, validating the long-term efficacy of these endoprostheses requires larger cohorts and extended follow-up periods [44]. Furthermore, a characteristic of 3D-printed hemipelvic endoprostheses is their lack of intraoperative adjustability and poor flexibility, necessitating bone resection guided by the cutting guide [28]. Particularly in patients with large soft tissue masses, achieving precise bone resection and proper fitting between the cutting guide and host bone can be challenging, leading to difficulties in intraoperative implantation [2]. Thus, the rational design of the cutting guide is crucial to ensure precise bone resection and adherence to the preoperative implantation plan. To the best of our knowledge, this study presents the most extensive follow-up on using 3D-printed custom hemipelvic prostheses in limb-savage surgery within this specific case cohort. It is also the first to categorize typical pelvic defect types where such prostheses were used, and comprehensively evaluate diverse prosthesis designs, surgical methods, cutting guide designs, and clinical outcomes. Notably, innovative osteotomy guide designs were introduced to ensure precise bone resection and accurate prosthesis implantation techniques. We aim to evaluate the precision of implantation and further assess the long-term clinical efficacy in achieving complete pelvic ring reconstruction after tumour resection.

## Materials and methods

### Patients involvement

We retrospectively analyzed the results of patients who underwent limb-salvage surgery and reconstruction with 3D-printed custom hemipelvic endoprostheses for the treatment of primary pelvic malignancies between January 2017 and May 2022. The inclusion and exclusion criteria of this study are as follows: Inclusion criteria: i) Pathologically confirmed primary pelvic malignancy, ii) En bloc resection feasibility, iii) Reconstruction with 3D-printed custom endoprostheses, iv) Complete follow-up data. Exclusion criteria: i) Unwillingness or inability to accept prosthetic risks, ii) Serious comorbidities incompatible with anesthaesia and surgery, iii) Active infection at implantation site, iv) Metal implant allergy, v) Lower limb deformities, vi) Severe osteoporosis, vii) Incomplete follow-up data.

A total of 96 patients meeting inclusion criteria were enrolled, comprising 52 males and 44 females, with a median age of 47.4 ± 16.9 years at surgery. Diagnoses included: Chondrosarcoma (49 patients), Osteosarcoma (28 patients), Ewing sarcoma (9 patients), Solitary plasmacytoma (2 patients), Synovial sarcoma (2 patients), Spindle cell carcinoma (1 patient), Myofibroblastic sarcoma (1 patient), Solitary fibrous tumour (1 patient), Invasive chondroblastoma (1 patient), Malignant rhabdoid tumor (1 patient), and Langerhans cell histiocytosis (1 patient). The mean follow-up duration was 48.1 ± 17.9 months (range, 6 to 76 months). Patient demographics are summarized in Table 2.

**Table 2:** Characteristics of patients undergoing hemipelvic replacement surgery

Characteristics	All patients (*N* = 96)
Demographic	
Sex*	
Male	52 (54.2%)
Female	44 (45.8%)
Age† (yr)	47.4 ± 16.9
BMI† (kg/m^2^)	24.4 ± 3.2
Follow-up time † (mo)	48.1 ± 17.9
Tumor histology*	
Chondrosarcoma	49 (52.0%)
Osteosarcoma	28 (29.2%)
Ewing sarcoma	9 (9.4%)
Solitary plasmacytoma	2 (2.1%)
Synovial sarcoma	2 (2.1%)
Spindle cell carcinomas	1 (1.0%)
Myofibroblastic sarcoma	1 (1.0%)
Solitary fibrous tumor	1 (1.0%)
Invasive chondroblastoma	1 (1.0%)
Malignant rhabdoid tumor	1 (1.0%)
Langerhans cell histiocytosis	1 (1.0%)
Tumour volume (Length x Width x Height, cm)	
Tumour length† (cm)	12.5 ± 4.2
Tumour width† (cm)	9.2 ± 3.7
Tumour height† (cm)	7.2 ± 5.1
Preoperative staging*	
IIB	82 (85.4%)
III	14 (14.6%)
Neoadjuvant chemotherapy	
No. of patients*	43 (44.8%)
Resection Type^a^	
Type I + II	44 (45.8%)
Type I + II (Intact OF)	23 (24.0%)
Type I + II (Non-intact OF)	21 (21.9%)
Type II + III	24 (25.0%)
Type II + III (Intact PS)	10 (10.4%)
Type II + III (Non-intact PS)	14 (14.6%)
Type I + II + III/I + II + III + IV	22 (22.9%)
Type III	6 (6.3%)

^a^According to Enneking-Dunham Classification system [45]

*The values are given as the number of patients, with the percentage in parentheses

†The values are given as the mean and the standard deviation

*OF* obturator formamen, *PS *pubic symphysis

Preoperatively, all patients underwent pathological examination for diagnosis and Enneking staging for tumour classification [46], along with plain radiography, 3D-CT, MRI, and SPECT for lesion evaluation. This study was performed in accordance with the 1964 Helsinki Declaration and was authorized by the Ethics Committee of our hospital. Written informed consent was obtained from adult participants or parents of minors (below 16 years of age).

### Classification and Custom Osteotomy Guide Design for 3D-Printed Hemipelvic Prostheses

**Fig. 1:**
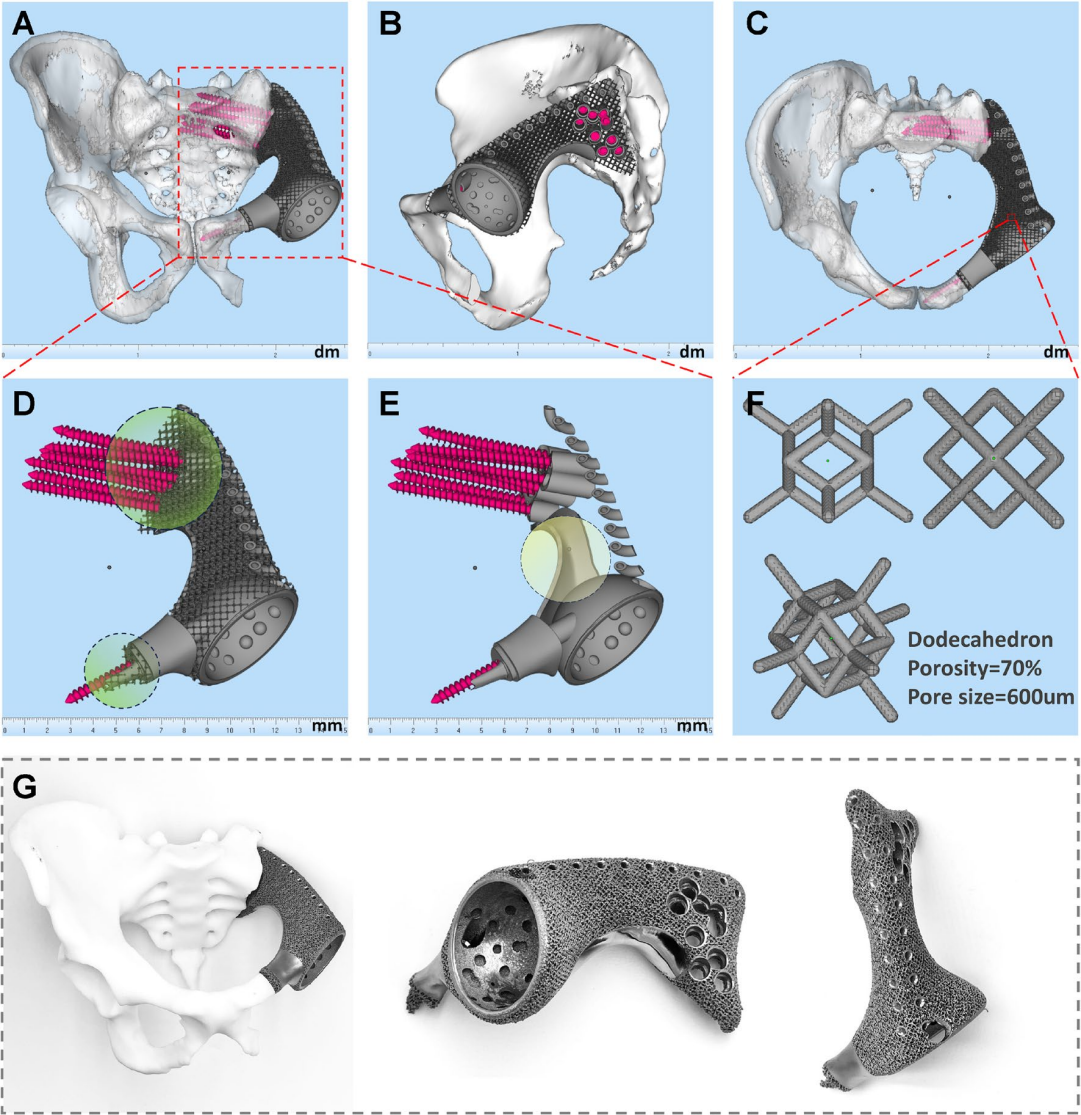
illustrates a 3D-printed hemipelvic prosthesis reconstruction for a 46-year-old male after type I + II resection: Views include frontal (**A**), lateral (**B**), and superior (**C**). The prosthesis (**D**) features a porous structure with circles denoting contact areas on sacroiliac and pubic bone surfaces, promoting osteointegration. Solid components (**E**) include screw paths, acetabular cup, pubic stem, and weight-bearing core (circled greater sciatic notch section), providing mechanical strength. The porous structure's microstructural unit (**F**) forms a 12-faced crystal lattice (Dodecahedron). Photographs (**G**) of the 3D-printed hemipelvic endoprosthesis align with computer design

**Fig. 2:**
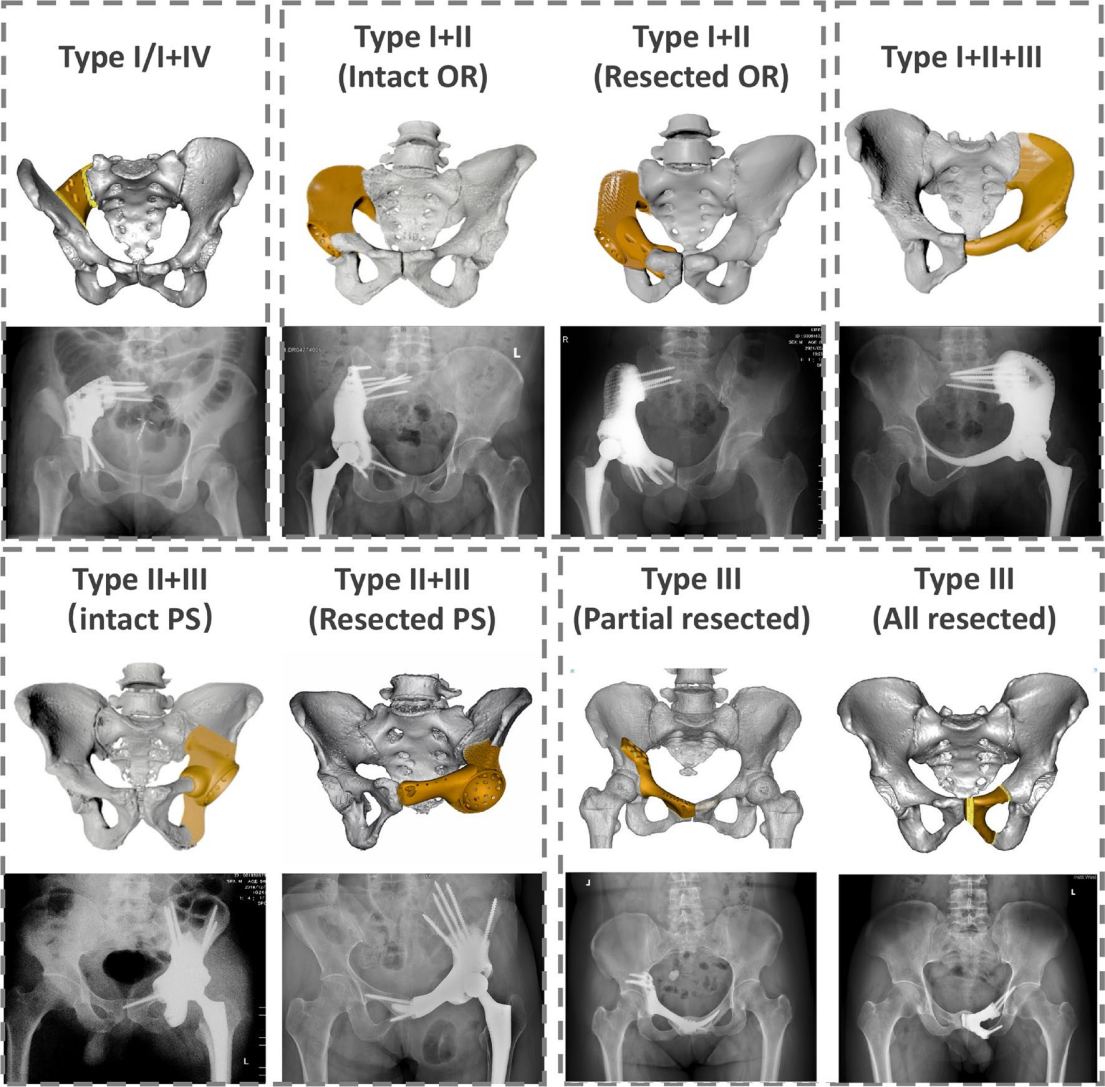
Classification of 3D-Printed Custom-Made Hemipelvic Prostheses: Based on the Enneking classification of pelvic resection types, the prostheses are categorized into five types

All endoprostheses were designed by our clinical team and fabricated by Chunli Co., Ltd. (Tongzhou, Beijing, China). Streamlined workflow for 3D-printed custom hemipelvic prostheses design followed our previous study [47]. (Fig. 1, Figure S1-2, Supplementary content 1, and Video 1–2, Supplementary content 2). Based on the Enneking classification system for pelvic tumor resections [46], we classify the prostheses into five types: i) Type I/I + IV resections; ii) Type I + II/I + II + IV resections, with subdivisions based on obturator ring preservation; iii) Type I + II + III/I + II + III + IV resections; iv) Type II + III resections, with further subclassifications based on pubic symphysis and ischial region preservation; v) Type III resections, with subtypes based on pubic symphysis extent. (Fig. 2 and Video 3, Supplementary content 2) Tumor resection scope, surgical approach selection, and key prosthesis design factors are summarized in Table 3.

**Table 3:** Bone Resection, Surgical Approaches, Implant Design, and Fixation Criteria for Prosthesis Types

Prosthesis Type	Resection scope^a^	Integrity of OR	Integrity of PS	Surgical position	Surgical approach	Screw quantity for stable prosthesis-bone fixation		Prosthesis design requirements
						Proximal interface	Distal interface	
1	Type I/I + IV	Intact	Intact	Lateral position	Posterior iliac incision with optional posterior longitudinal midline extension when needed	≧ 4	≧ 3	Restore the lumbosacral-iliac joint-pelvic connection
2	Type III	Incomplete	Incomplete	Oblique supine lithotomy position	Ilioinguinal incision	≧ 3	Stem fixation	① Restore anterior pelvic ring; ② Effective pubic symphysis fixation
3	Type I + II	Intact/ Incomplete	Intact	Lateral position	Combined posterior iliac and Smith-Petersen approaches, optionally incorporating the ilioinguinal approach	≧ 4	≧ 2	① Restore hip joint function; ② Effective sacroiliac joint fixation; ③ Effective pubic symphysis fixation
4	Type II + III	Incomplete	Incomplete	Lateral position	MGMII approach			
5	Type I + II + III/I + II + III + IV	Incomplete	Incomplete	Lateral position	MGMII approach	≧ 4	Stem fixation	① Restore hip joint function; ② Effective sacroiliac joint fixation; ③ Effective pubic symphysis fixation

^a^According to Enneking and Dunham Classification [45]

*SI *sacroiliac joint, *OR *obturator formamen, *PS *pubic symphysis, *MGMII *combined and modified Gibson and ilioinguinal approach

**Fig. 3:**
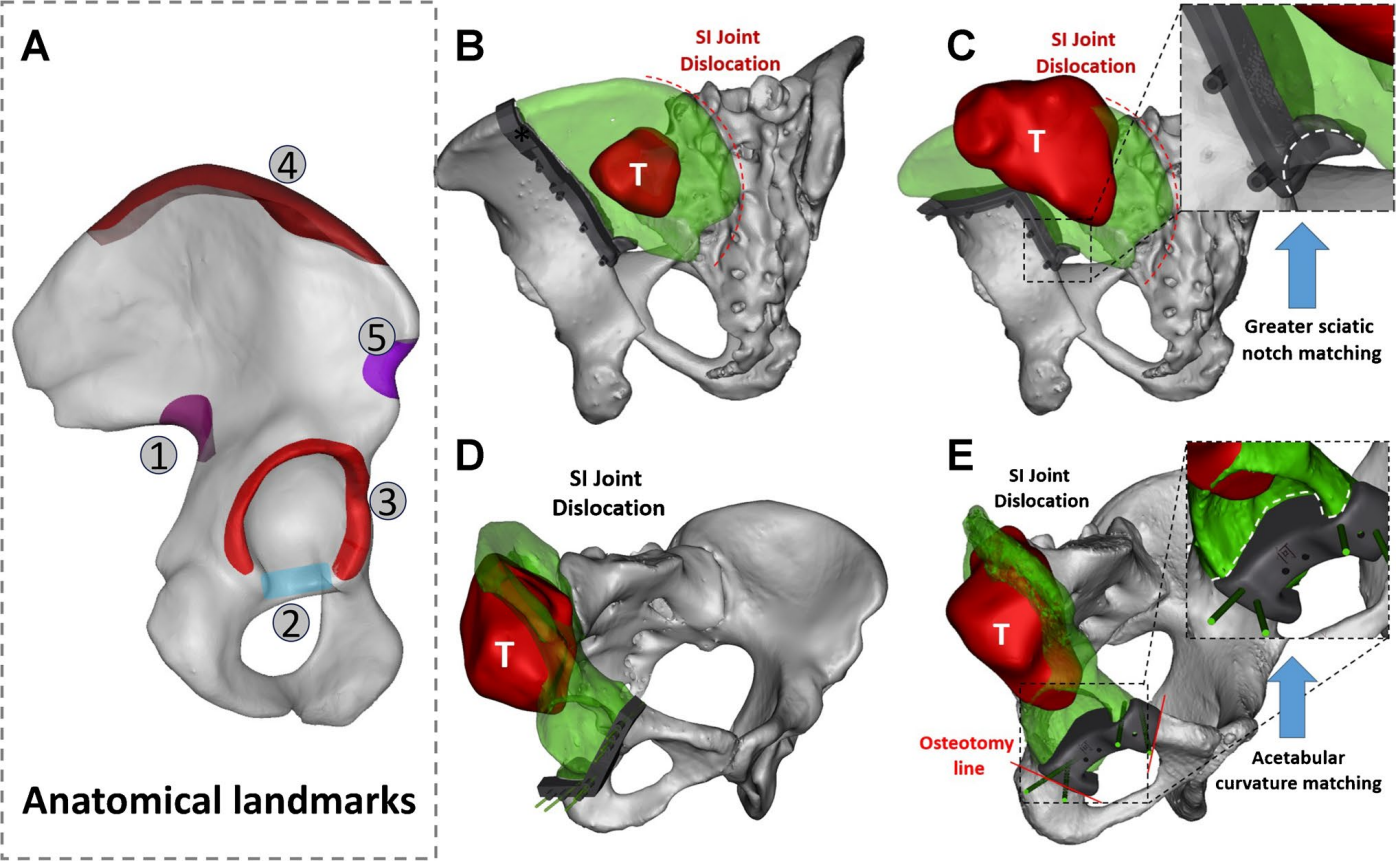
Customized Cutting guide design for periacetabular tumour: **(A)** Hemipelvic replacement surgery utilizes anatomical landmarks such as ① the greater sciatic notch, ② acetabular notch, ③ acetabulum rim, ④ iliac crest, and ⑤ iliac spines as anchor points for cutting guides. (**B**) Proximal unilateral osteotomy; (**C**) Proximal bilateral osteotomy. (**D**) Distal osteotomy with preserved obturator foramen; (**E**) Distal osteotomy without preserved obturator foramen. In the illustrations, red denotes the tumor (T), and green indicates areas of pelvic resection, all depicting cases with sacroiliac joint separation

Furthermore, we compiled a catalog of pelvic landmarks for precise osteotomy guide plate positioning, using identifiable irregular landmarks as anchor points. Kirschner wires are used to securely fix the cutting guide in place (Figure S3, Supplementary content 1). Osteotomy areas are categorized into anterior and posterior pelvic regions (Fig. 3A and Table 4). The expansive iliac crest and lower greater sciatic notch serve as anchor points in the posterior pelvic region (Fig. 3B-C), while the acetabulum is used in the anterior pelvic region (Fig. 3D-E and Video 4, Supplementary content 2).

**Table 4:** Selection of Anchoring Points and Design Details of Bone Resection Guides on the Pelvis

Anchoring points	Anatomical landmarks	Anchoring requirements	Relevant surrounding blood vessels and nerves	Surgical site exposure requirements
1	Greater sciatic notch	Secure irregular notch, ≥ 15 mm contact width	Sciatic nerve	/
2	Acetabular notch	Fill irregular acetabular notch shape	/	Femoral head dislocation
3	Acetabulum rim	Hook acetabular contour/partially fill acetabular notch	Anterior acetabular rim: Femoral artery and vein, and femoral nerve	Femoral head dislocation/debridement of cartilage structures
4	Iliac crest	Hook the iliac crest edge	/	/
5	Anatomical notches at the anterior superior and inferior iliac spines	Hook the iliac crest edge	Femoral lateral cutaneous nerve (LFCN)	/

### Surgical procedures and postoperative management

The surgical procedures were performed by two experienced surgeons. Tumour resections were en-bloc, guided by preoperative simulations to expose bone adequately for precise placement of patient-specific instruments. Custom cutting guides were secured with 2 mm K-wires, and osteotomies were performed meticulously using an ultrasonic bone scalpel. Post-implantation, we cleaned wounds with 10% povidone-iodine for 3 min followed by pulsatile lavage using isotonic sodium chloride solution. Prosthesis fixation primarily relied on cancellous bone screws, typically starting at the proximal end, especially for Zone IV resections. We exposed the sacral trabecular bone, implanted the porous-surfaced prosthesis, and inserted screws along planned paths from proximal to distal. If pubic bone insertion was required, we considered a stem with a porous surface design. Screws were used to secure the ischial region if preserved. For hip joint replacement, we cemented a constrained acetabular liner with a slight 5° to 10° deviation from natural anteversion. Following this, we meticulously implanted proximal femoral components and delicately reconstructed the preserved muscles and their origins.

After surgery, lower limb immobilization was maintained in specific positions (neutral rotation, 15° to 25° hip abduction, 15° hip flexion, and 15° knee flexion). All patients underwent personalized postoperative rehabilitation training plans based on previous reports from our center [45]. Clinical and radiological evaluations were systematically performed at regular intervals post-surgery: initially at one, two and three months, followed by assessments every three months for the first two years, and subsequently every six months. These evaluations encompassed various aspects, including i) oncological outcomes; ii) function assessments utilizing MSTS-93 scale, along with assessment and recording of patient Range of Motion (ROM), Limb Length Discrepancy (LLD), as well as walking and weight-bearing capacities; iii) surgical outcomes such as operation duration and blood loss; iv) Pain control assessment using VAS Scale; v) Evaluation of complications; and vi) Radiological analysis of osteointegration using Tomosynthesis Shimadzu Metal Artefact Reduction Technology (T-SMAR) for all patients [28, 31, 36, 45, 48, 49].

### Statistical analysis

Independent-samples Student’s t-test for normally distributed data (operating time, intraoperative blood loss, VAS score, MSTS93 functional score). Mann–Whitney U test for non-normally distributed data. SPSS 21.0 used for analysis (IBM Corp., Armonk, NY), and Prism software (GraphPad, La Jolla, CA) for graphical presentation. *p* < 0.05 (two-tailed test) considered statistically significant.

## Results

### Oncological outcomes and functional assessments

**Fig. 4:**
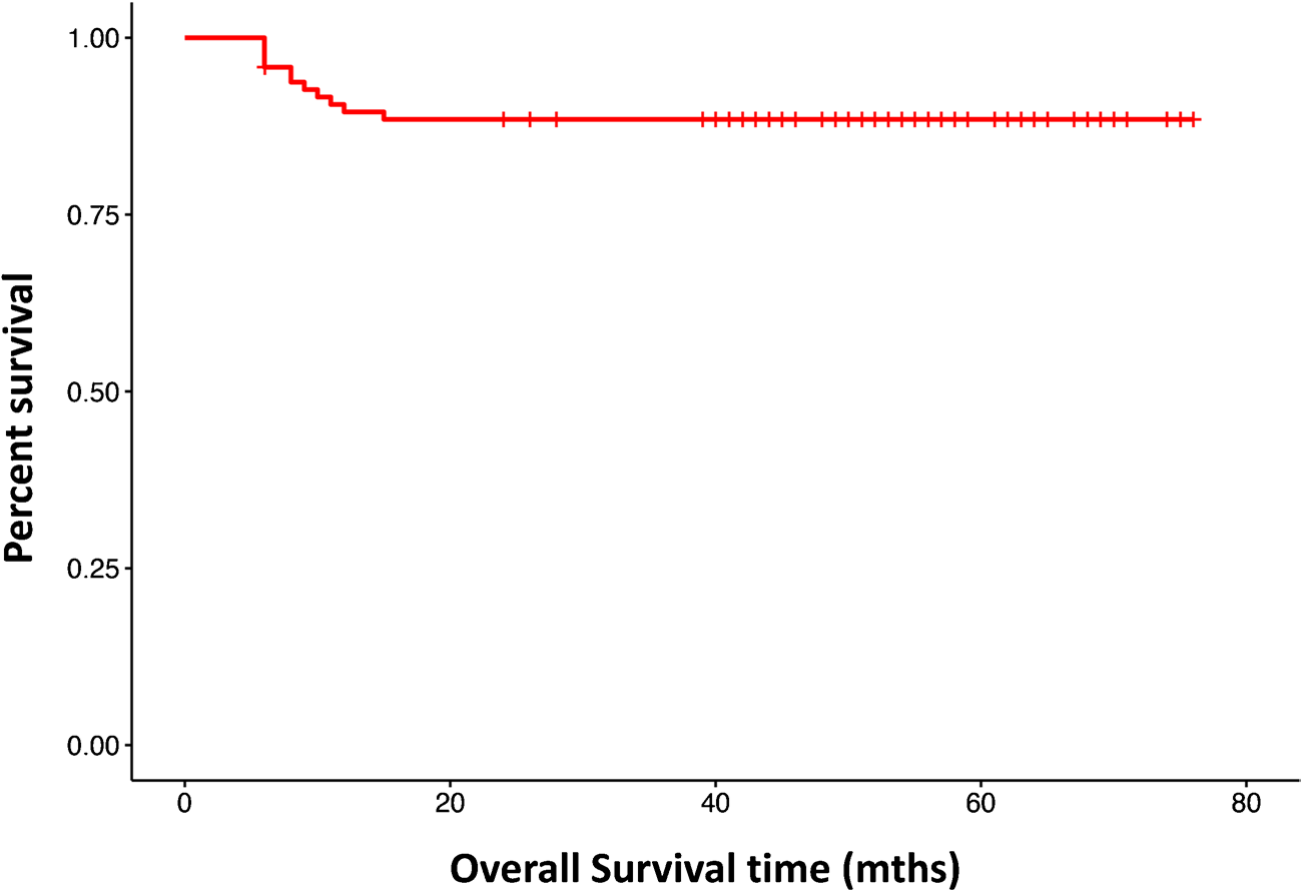
Overall survival curve of 96 patients with primary malignant pelvic tumors undergoing En Bloc resection and 3D-printed custom hemipelvic endoprosthesis reconstruction

Regarding oncological outcomes, at the latest follow-up, 70 patients (73.0%) sustained survival without evidence of disease, 15 (15.6%) were alive with disease, and 11 (11.4%) succumbed to metastatic disease, demonstrating an average postoperative survival time of 8.8 ± 3.0 months (ranging from 6 to 15) (Fig. 4).

For functional outcomes, patients experienced satisfactory postoperative function. MSTS-93 score improved from 12.2 ± 2.0 points (8 to 16) preoperatively to 23.8 ± 3.8 points (4 to 29) at the most recent follow-up. Furthermore, except for the four patients who underwent amputation due to uncontrollable infection, all patients could sit cross-legged and had good squatting function. They were able to walk continuously for a median duration of 1.2 ± 0.4 h (range 0.5 to 2.3 h) and achieved good weight-bearing capability. Regarding hip range of motion (ROM), these patients had an average hip flexion of 104.4° ± 7.2 (range 90–120°) and an average hip extension of -7.1° ± 6.1 (-20° to 0°). Additionally, the mean limb length discrepancy (LLD) was 1.3 ± 1.0 cm (range 0.0–3.5 cm).

### Surgical outcomes and pain control assessment

The application of 3D-printed hemipelvic prostheses combined with customized osteotomy guides resulted in relatively short operation times and acceptable intraoperative blood loss control. En bloc resection was accomplished in all patients. The mean total surgical duration, from incision to wound closure, was 275.1 ± 94.0 min (range: 170–680.0 min). Intraoperatively, the mean blood loss amounted to 1896.9 ± 801.1 ml (range: 500.0–6000.0 ml). In terms of pain control assessment, the VAS score improved significantly from 5.3 ± 1.8 points (2 to 8) preoperatively to 1.4 ± 1.1 points (0 to 6) at the latest follow-up.

### Complications

Intraoperatively, one patient suffered bladder and ureter damage, causing urinary leakage into the abdomen. To prevent prosthesis-related infection, pelvic reconstruction was omitted. In addition, postoperative complications affected 13 patients (13.5%), with no notable difference in the overall complication rate among prosthesis subtypes. The most common complication was poor wound healing in six patients (6.3%). Among them, two cases were successfully managed with intensive wound dressings, while the remaining four required debridements and Vacuum Assisted Closure (VAC) drainage. Deep prosthesis infections afflicted four cases (4.2%), despite the diligent application of sustained Debridement, Antibiotics, and Implant Retention (DAIR) procedures. However, the infections persisted and proved refractory, ultimately requiring the eventual removal of the implant and hemipelvectomy as a last resort to achieve infection control. Two cases experienced postoperative hip dislocation (2.1%) on the second day due to inappropriate positioning of the affected limb. These were resolved through closed reduction under anaesthesia and subsequent stabilization using a T-shaped pillow and anti-rotation shoes, effectively preventing further dislocation. In one case of Type II + III resection with reconstruction, there was an upper sacroiliac joint screw fracture (1.0%) post-surgery, although the patient remained asymptomatic without affecting the prosthesis or limb function. Another patient with a Type II + III resection and reconstruction exhibited distal-bone interface loosening (1.0%) and fractures in pubic and ischial screws after a two year follow-up. A subsequent revision surgery significantly improved lower limb function.

### Radiographic outcomes

**Fig. 5:**
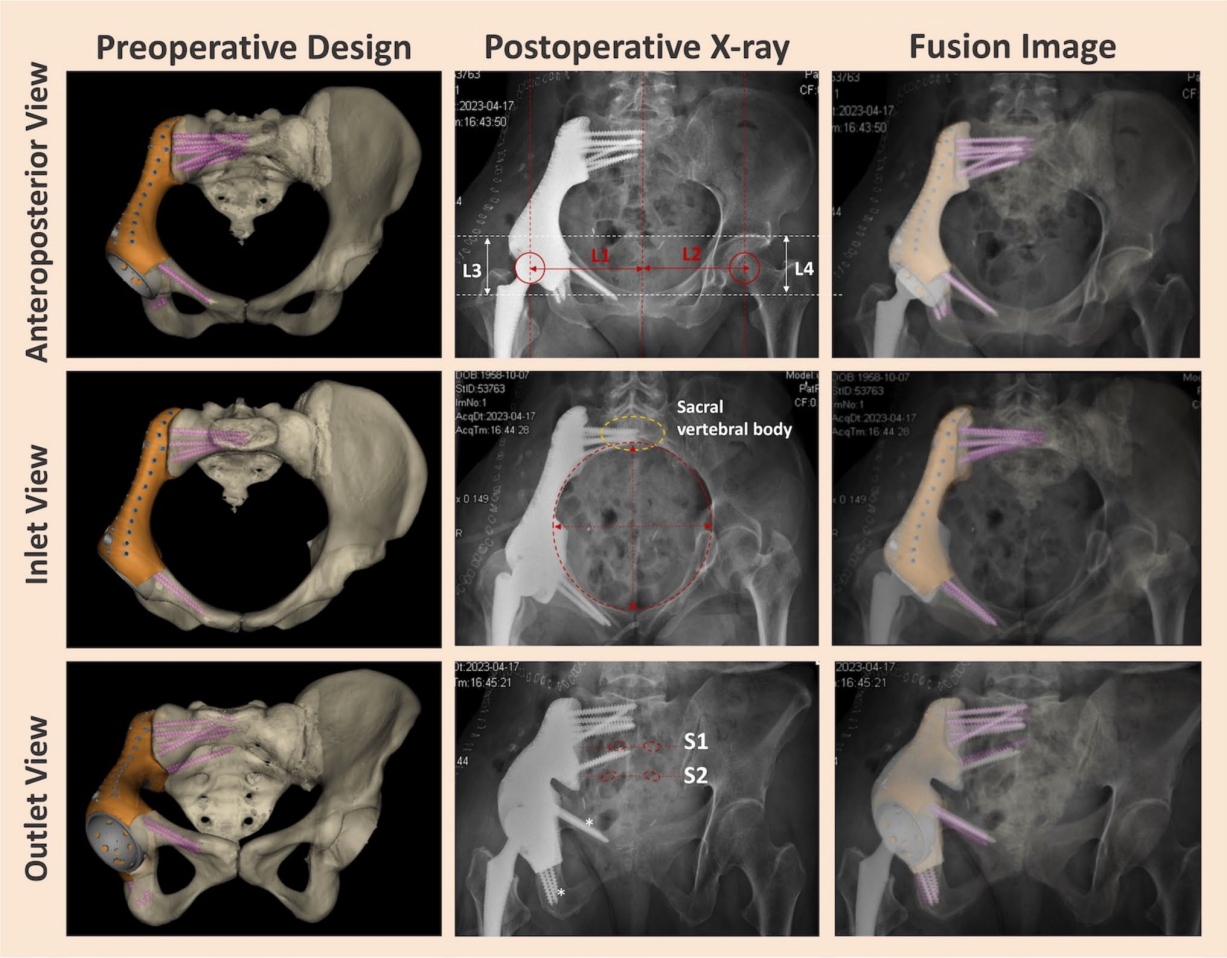
A typical case of 3D-printed custom hemipelvic prosthesis reconstruction: Postoperative pelvic X-ray evaluation confirms precise implantation of the 3D-printed custom hemipelvic prosthesis according to the preoperative plan, including accurate screw placement and prosthesis positioning. Anteroposterior views show the hip joint rotation centre (red circle) and femoral head eccentricity in both horizontal (L1 = L2) and vertical (L3 = L4) directions. Inlet views display sacroiliac joint screw placement, avoiding vertebral canal encroachment (yellow circle). Small pelvic ring reconstruction is evident (red dashed circle), and outlet views show accurate sacroiliac joint screw placement, taking care to avoid the S1/S2 sacral foramina (red dashed box). Ischium and pubic bone screws provide stability and support (Reprinted with permission from Hu et al. ©2024 Journal of Orthopaedic Surgery and Research [50])

All patients underwent precise osteotomy, accurate prosthesis implantation, and planned screw fixation (position, quantity, and direction) consistent with the preoperative plan. In addition, except for one Type II + III resection patient experiencing distal prosthesis loosening, all other patients exhibited successful osseointegration of their implants during the final follow-up examination (T-SMART). Postoperative X-ray examinations revealed no evidence of bone absorption or osteolysis at the prosthesis-bone interface. In Fig. 5, we showcased an illustrative example depicting the precise implantation of prosthetics according to the preoperative plan.

## Discussion

Pelvic bone tumor resection and the subsequent pelvic reconstruction are technically demanding and challenging due to complex anatomy, load-bearing demands, and significant defects [2, 17–19]. While hindquarter amputation was previously common, recent advancements have led to improved methods for limb preservation and reconstruction [2]. Notably, 3D-printed hemipelvic prostheses offer anatomically matched solutions for bone defects and promote bone integration, gaining popularity among orthopedic surgeons. In this study, we developed 3D-printed custom hemipelvic endoprostheses for pelvic girdle reconstruction following bone tumor resections. Through extensive clinical follow-up of 96 patients, with an average follow-up period of 48.1 ± 17.9 months, we observed favorable clinical outcomes, evidenced by an average MSTS-93 score of 79.3%, alongside reduced incidences of postoperative infections and prosthesis loosening compared to similar studies [1, 11, 12, 17, 18, 51].

Our study's primary limitation is the heterogeneity of pathohistological types among pelvic tumours, which may introduce bias and influence postoperative oncological outcomes. However, this diversity allowed for a broader patient population with pelvic malignancies, enabling a more precise evaluation of the long-term clinical effectiveness of 3D-printed custom hemipelvic prostheses. Moreover, the use of 3D-printed custom prostheses in orthopaedics presents challenges like prolonged design and production timelines, potentially increasing the risk of tumour progression [17, 18, 22, 29–33]. Thus, establishing a standardized protocol for prosthesis design and implantation is crucial for improving clinical outcomes. Furthermore, the Integration of Augmented Reality in pelvic sarcoma surgeries shows promising potential and may soon optimize 3D-printed hemipelvic prosthetic reconstruction techniques [52].

The primary outcomes of this study are oncological and functional outcomes. In terms of oncological outcomes, the most important factor determining the risk of postoperative recurrence in pelvic malignant tumors is whether sufficient surgical margins can be achieved. In this study, precise tumor resection within predetermined surgical margins was achieved by preoperative computer virtual surgery, combined with patient imaging data. We determined these margins based on previous studies, which indicated a median tumor-free bone resection margin of 10 mm for chondrosarcoma. However, for high-grade sarcoma patients without effective preoperative treatment, a 30-mm tumor-free bone resection margin was considered adequate; with effective preoperative treatment, a 20-mm margin sufficed. Based on the latest follow-up results, the risk of local recurrence in patients in this study closely resembles that in similar studies [17, 22, 30, 33, 34]. 3D-printed customized hemipelvic prosthetic reconstruction does not compromise surgical margins. Hence, its efficacy in tumour control is comparable to traditional reconstruction methods.

**Fig. 6:**
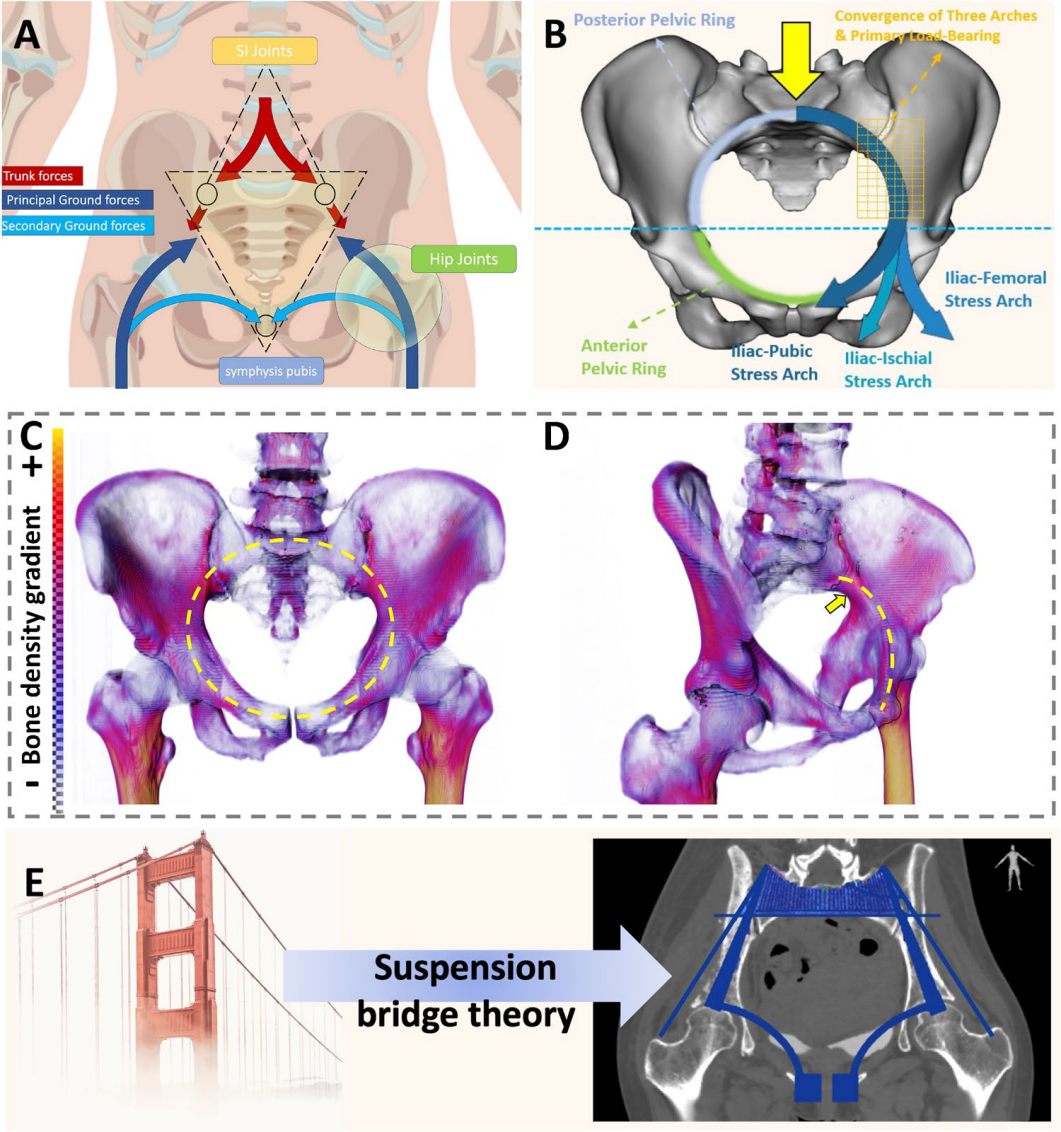
Stress transmission patterns in a normal pelvic girdle and the biomechanical significance of complete pelvic girdle reconstruction: (**A**) Diagram of stress transmission in the healthy pelvis (Reprinted with permission from Hu et al*.* ©2023 British Journal of Surgery [28]). (**B**) The posterior pelvic ring bears the primary weight, particularly at the intersection of three stress arches (iliac-femoral, iliac-pubic, and iliac-ischial stresses, shown in the yellow grid area), while the anterior pelvic ring supports secondary load-bearing to aid stress distribution and overall stability. (**C**-**D**) 3D pelvis model from a 46-year-old male volunteer's CT data using Thermo Scientific Avizo software (ThermoFisher Scientific, Waltham, MA, USA). The yellow dashed circle highlights a core weight-bearing area with relatively high bone density in the pelvic girdle, while the yellow arrow in (**D**) points to the highest bone density in the ischial foramen region. (**E**) In the suspension bridge analogy, the posterior superior iliac spines act as pillars, the interosseous sacroiliac ligaments are the suspension cables, and the sacrum serves as the central bridge, supported by the anterior pelvic ring for stability (Reprinted with permission from Unsplash and Photo by Stephen Leonardi)

For functional outcomes, the achievement of satisfactory results can be attributed to four key factors: i) Precise Implantation: The use of custom-designed prostheses and cutting guides ensures an anatomically precise fit, overcoming challenges associated with aligning modular pelvic prostheses with diverse defects [11, 53]. ii) Secure Fixation: Effective prosthesis fixation promotes initial stability, crucial for osseointegration and preventing early aseptic loosening [41]. Our prosthetic stability is achieved using multiple cancellous bone screws and a pubic stem, with screw directions based on preoperative simulations to align with stress transmission. iii) Complete Pelvic Ring Reconstruction: In contrast to incomplete pelvic girdle reconstruction [29, 39, 40, 54], complete restoration of the pelvic ring aligns with natural stress distribution [30, 32, 34, 36, 42, 55, 56], mitigating stress discontinuities and reducing the risk of mechanical failure [45]. (Fig. 6A-D) iv) Effective Osseointegration: Effective osseointegration at the prosthesis-bone interface is a vital condition for long-term prosthesis survival [32, 36, 57, 58]. We incorporated a porous structure mimicking trabecular bone at the interface where the prosthesis contacts the bone. Moreover, autografts from non-tumor areas (e.g., femoral head) were used near the implant-bone connection, further enhancing osseointegration [36]. T-SMART Results confirmed that a vast majority of patients achieved effective prosthesis-bone interface integration.

The study’s secondary outcomes comprised surgical duration, intraoperative bleeding, pain control, and complications. Implementing the streamlined prosthetic reconstruction process proposed in this study facilitated efficient surgical timing and intraoperative bleeding control. Preoperative 3D computer modeling and image fusion techniques allowed for anticipation of surgical complexity. Leveraging distinct anatomical landmarks in the pelvis as secure anchor points, along with precise matching of osteotomy guides, expedited the bone resection phase. This approach facilitated swift osteotomy and accurate implantation of prostheses using pre-customized cutting guides and planned screw fixation. These strategies collectively reduced surgical time, with an average duration of 275.1 ± 94.0 min, lower than reported in comparable studies on 3D-printed custom prosthetic reconstruction after en bloc resection of pelvic bone tumors (typically ranging from 234 to 618 min) [1, 17, 18, 23, 29, 38, 43, 59–61]. Furthermore, a benefit accompanying the reduction in surgical time is a decrease in overall intraoperative blood loss. In this study, the observed average intraoperative blood loss was 1896.9 ± 801.1 ml, lower than the reported blood loss in similar studies of 3D-printed hemipelvic prosthetic reconstruction of the pelvic girdle after tumour resection [29, 32, 55, 62].

Infections and aseptic loosening of prostheses are widely recognized as the most common causes of long-term failure following hemipelvic prosthesis replacement surgery [17, 19, 22, 63, 64]. In our study, the combined reduction in surgical time and intraoperative blood loss, coupled with empirically guided infection prevention measures, may contribute to the observed lower infection rates. These preventive measures included repetitive pulsed lavage and povidone-iodine soaking of the wound, alongside a simplified design for custom prostheses that eliminates unnecessary parts while retaining essential weight-bearing structures, which has successfully decreased prosthesis volume [49]. This approach enables superior soft tissue coverage under identical conditions, eliminating potential dead spaces in the pelvic region. In addition to reducing the risk of postoperative infection, we also observed a low incidence of postoperative loosening during mid-term follow-up, attributed to the achievement of stable prosthesis fixation and effective bone integration at the prosthesis-host bone interface.

## Conclusion

Our streamlined workflow for 3D-printed custom hemipelvic prosthetic reconstruction, complemented by tailored cutting guide design, offers potential benefits such as precise osteotomy according to preoperative planning, accurate implantation of the prosthesis, and initial stable fixation. Furthermore, integrating biomimetic porous structures and in-surgery autografts may enhance long-term osseointegration at the prosthesis-host bone interface, thereby mitigating the risk of loosening. Consequently, 3D-printed custom hemipelvic prostheses present a promising alternative for pelvic girdle reconstruction following tumour resections.

### Supplementary Information

Below is the link to the electronic supplementary material.

Supplementary file1 (DOCX 15386 KB)

Supplementary file2 (PPTX 117671 KB)

## Data Availability

The datasets used and/or analysed during the current study are available from the corresponding author on reasonable request.
