# Supplementary material for: Advanced Pelvic Girdle Reconstruction with three dimensional-printed Custom Hemipelvic Endoprostheses following Pelvic Tumour Resection
Source: Int Orthop. 2024 May 22;48(8):2217–31. doi: 10.1007/s00264-024-06207-3 (PMC11246265; doi:10.1007/s00264-024-06207-3)
Supplement: Supplementary file 1 — Supplementary file1 (DOCX 15386 KB) [file 264_2024_6207_MOESM1_ESM.docx]

**Advanced Pelvic Girdle Reconstruction with 3D-printed Custom Hemipelvic Endoprostheses following Pelvic Tumor Resection**

SUPPLEMENTARY MATERIAL

Figures

**
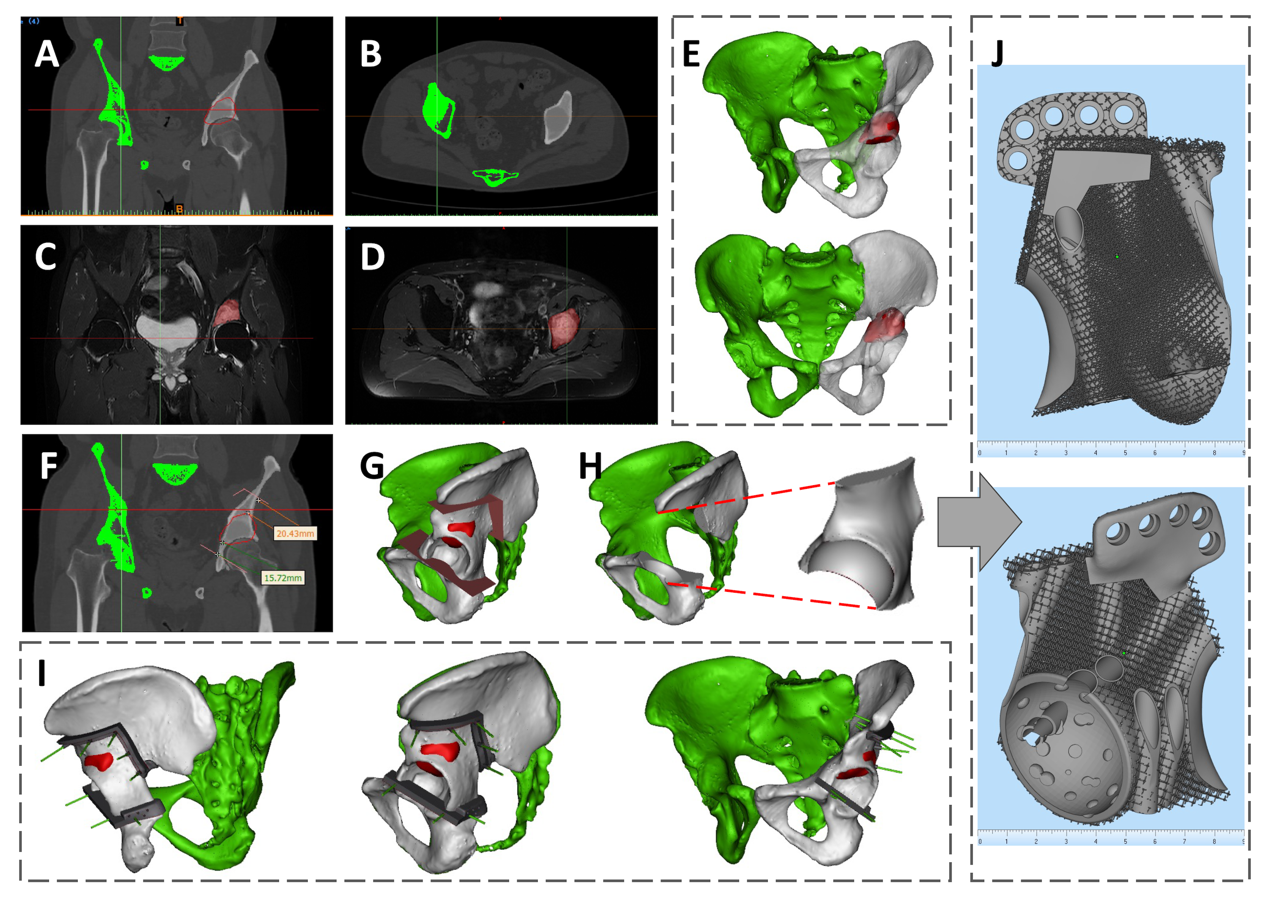
**

**Figure S1. 3D-printed Custom Prosthesis Design and Production Process:** Employing Mimics software, we reconstructed a three-dimensional model of the pelvis and tumor using preoperative three-dimensional CT and MR data from a 53-year-old male patient diagnosed with a pelvic bone tumor. **(A-B)** The healthy side (in green) and affected side (in white) of the pelvis were reconstructed utilizing the "threshold segmentation" functionality within 3D CT imaging. **(C-D)** The tumor region (in red) is reconstructed based on the patient's pelvic MR data.

**(E)** The resulting 3D model of the pelvis and tumor. **(F)** Resection boundaries are determined on the 3D reconstruction model, considering the tumor's characteristics and extent of invasion. **(G)** The osteotomy region. **(H)** The bone fragments resulting from the osteotomy form the initial shape of the prosthesis, preserving the original anatomical morphology **(I)** Designing resection templates to match the surgical approach.


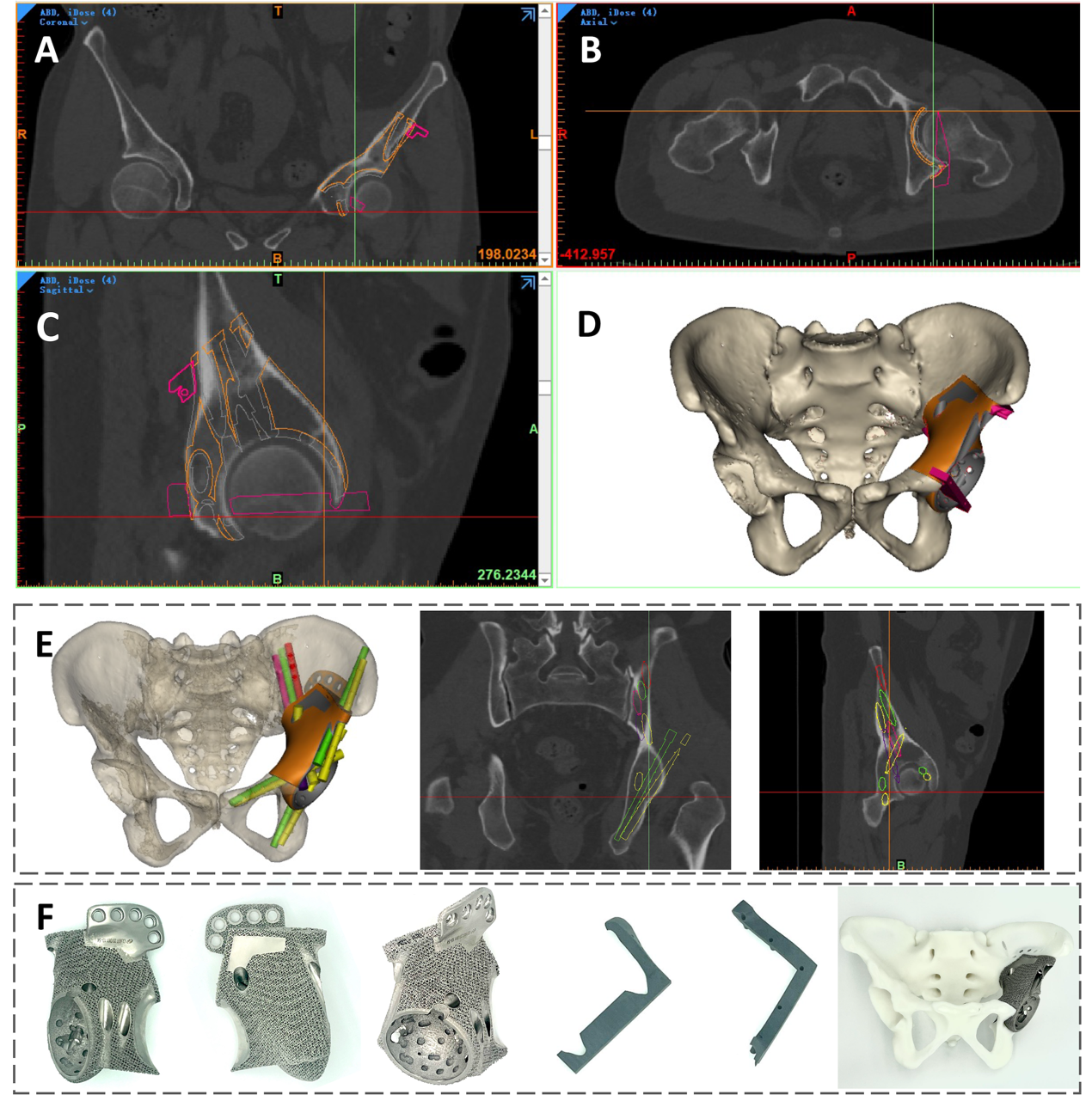


**Figure S2.** **3D-printed Custom Prosthesis Design and Production Process:** **(A-C)** Reimport the 3D models of the designed prosthesis and cutting guide into the patient's CT data. Reassess the compatibility of the prosthesis with the pelvis by overlaying the prosthesis and custom cutting guide outlines. **(D)** Simulate the installation of the cutting guide after bone resection and the implantation of the prosthesis. **(E)** Simulate the insertion of screws during the surgery. Based on the 3D CT results, adjust the direction and arrangement of the screws to ensure mechanical transmission, safe positioning, and sufficient bone for fixation. **(F)** Complete the 3D printing of the custom prosthesis, resection templates, and pelvic plastic model. The plastic pelvic model, after sterilization, is brought into the operating room to facilitate reference and measurement of relevant data during prosthesis implantation."


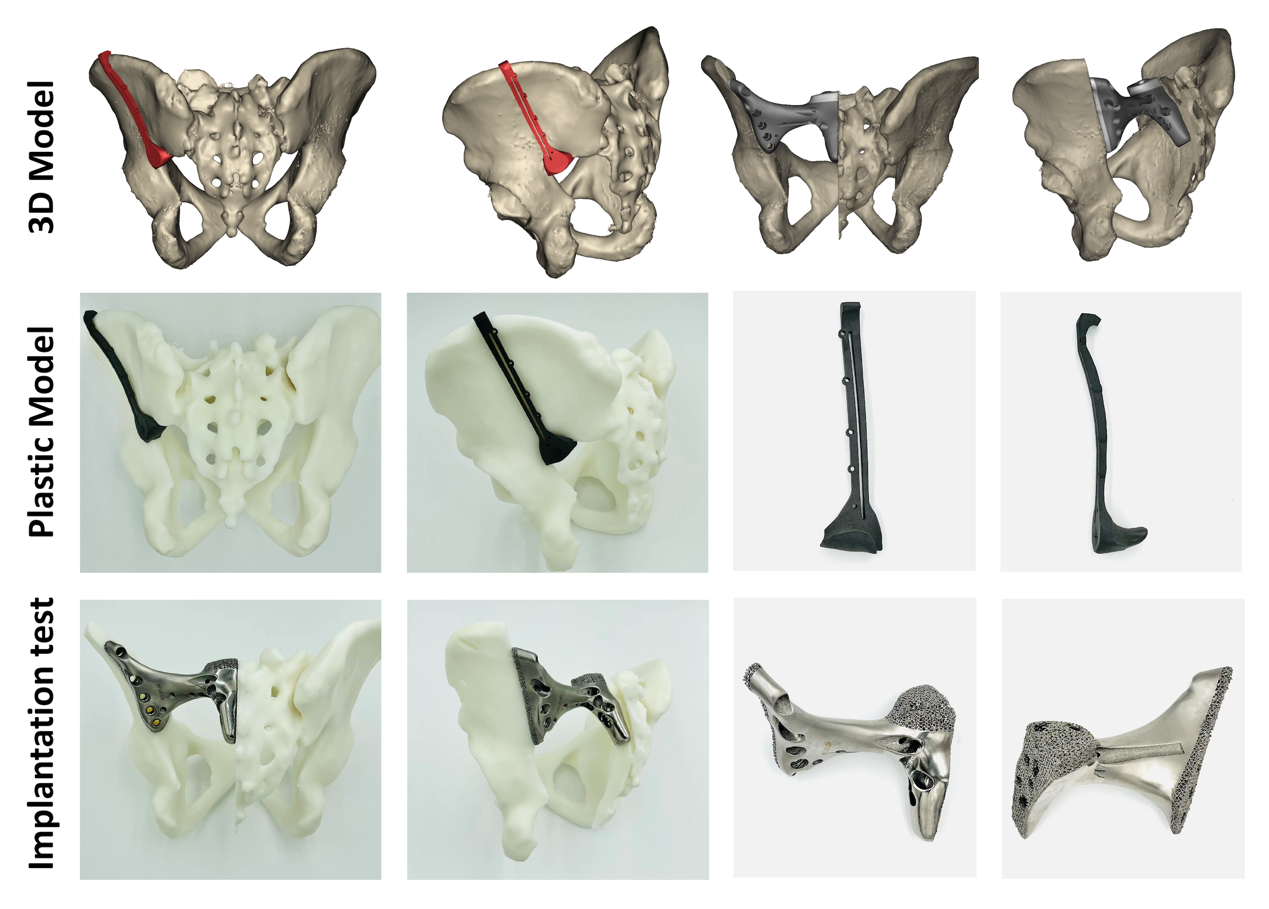


**Figure S3. Preoperative Accuracy Assessment of 3D-Printed Custom-Made Hemipelvic Endoprosthesis:** Based on 3D CT data of the affected limb's pelvis, a pelvic model is reconstructed. A plastic physical model is printed, and preoperative simulated resection and prosthesis installation are performed to confirm the prosthetic alignment with the planned design.
